# Redundancy cancellation of compressed measurements by QRS complex alignment

**DOI:** 10.1371/journal.pone.0262219

**Published:** 2022-02-08

**Authors:** Fahimeh Nasimi, Mohammad Reza Khayyambashi, Naser Movahhedinia

**Affiliations:** Faculty of Computer Engineering, University of Isfahan, Isfahan, Iran; Effat University, SAUDI ARABIA

## Abstract

The demand for long-term continuous care has led healthcare experts to focus on development challenges. On-chip energy consumption as a key challenge can be addressed by data reduction techniques. In this paper, the pseudo periodic nature of ElectroCardioGram(ECG) signals has been used to completely remove redundancy from frames. Compressing aligned QRS complexes by Compressed Sensing (CS), result in highly redundant measurement vectors. By removing this redundancy, a high cluster of near zero samples is gained. The efficiency of the proposed algorithm is assessed using the standard MIT-BIH database. The results indicate that by aligning ECG frames, the proposed technique can achieve superior reconstruction quality compared to state-of-the-art techniques for all compression ratios. This study proves that by aligning ECG frames with a 0.05% unaligned frame rate(R-peak detection error), more compression could be gained for *PRD* > 5% when 5-bit non-uniform quantizer is used. Furthermore, analysis done on power consumption of the proposed technique, indicates that a very good recovery performance can be gained by only consuming 4.9*μW* more energy per frame compared to traditional CS.

## Introduction

Real-time remote tracking of physiological signals such as ElectroCardioGram(ECG) has become an important topic in wireless healthcare [1]. Realization of long-term ubiquitous monitoring confronts multiple challenges, such as device size, cost and computational efficiency [2]. However, the major problem is the considerable amount of data to be aggregated and the limited battery life of sensors [3]. Studies have confirmed that sensors consume energy mostly through data transmissions [4]. So a variety of data reduction techniques assorted into lossy and lossless techniques can be used to improve the energy efficiency of sensors. Lossless compression techniques, as its name implies, reduces the amount of transmitted data without affecting the quality of the signal. In these methods, intra-beat redundancies existent in the frames are removed. With lossless methods, a limited amount of compression is gained compared to lossy methods. Lossy signal compression methods are segregated into three distinct techniques with their own advantages and disadvantages: Direct, transform and parameter extraction methods. The first two methods extract redundancy from the main domain and transform domain respectively and the former one extracts the parameters from an ECG signal and transmits them. Lossy methods are not suitable for many clinical applications because of the lossy nature. So In 2006, Donoho [5] proposed a compression method(transform method) called Compressed Sensing(CS) that solves mathematical algorithms for data reconstruction on the server from a received linear projection of a sparse or compressible signal with a random sensing matrix at the sensor. Despite the performance improvements made by CS techniques, they still have a persistent gap from the most beneficial transform-based compression methods such as Set Partitioning in Hierarchical Trees(SPIHT) when energy-based distortion metrics such as Percent Root-mean-square Difference (PRD) are considered. This motivates interest in new CS-based approaches that will improve performance in terms of signal quality.

The core of this paper is aligning frames in order to make use of its existence similarity to remove redundancy from measurement vectors. Authors previous work also focuses on removing redundancy from similar frames but in this study, the idea of using high similarity between aligned ECG frames is utilized for redundancy removal of measurement vectors. The proposed method aligns ECG frames according to the detected peaks, so after CS, there would be high redundancy available in measurements. Removing this redundancy results in nonuniform distribution of values with zero mean.

In order to study the ability of this technique for improving the performance of CS-based ECG signal compression, it was tested over various ECG records at different compression ratios. The experimental outcome of this study proves that the added stages to the plain CS increase sensors processing time a little, with the advantage of reducing the number of bits per sample in each frame and superior efficiency over various compression ratios. Despite the performance improvements made by the proposed techniques, very noisy ECG signals which make the R-peak detection technique error prone, remains challenging.

The unique contributions made in this work than the existing state-of-art research are:

- Aligning ECG segments in order to increase their existing similarity
- Framing each heart-beat
- Applying CS to each frame
- Using 5-bit quantizer

In the rest of this paper, after a brief literature review a description of CS, ECG signals and dictionary learning is given in **background** section. **Proposed scheme** section describes the proposed work.**Simulation and results** section presents an evaluation of the proposed work and compares it with several state-of-the-art techniques.

## Related work

The energy consumption of physiological sensors, particularly ECG sensors, has always been a challenging issue, so a variety of lossy and lossless techniques that reduce the energy expended in the transmission of ECG frames have been introduced. Lossless compression techniques, mostly extract static redundancies existence in the signal to reduce the total bit length. Codebook-based approaches are a popular format of these techniques, where values according to their frequency of occurrence, are assigned a short or longer binary code word [6]. Techniques, including Arithmetic or Huffman coding and Lempel-Ziv (LZ), are examples of lossless techniques [7]. In addition to dictionary-based implementations, Li et al. managed to classify real-time ECG waveform into four regions and use an adaptive prediction method for different regions. Later in order to simplify the transmit format, they used a modified variable length code to encode the prediction difference [8]. The work done in [9, 10] are also prediction-based lossless methods. The former work consists of an adaptive predictor based on fuzzy decision control, and the later one uses a linear slope predictor for data compression and incorporates a novel low-complexity dynamic coding-packaging scheme. Block-sorting techniques proposed by Arnavut et al. are also lossless ECG compression techniques which made use of Burrows-Wheeler Transformation and Inversion Ranks of Linear Prediction for ECG compression [11]. In terms of data compression performance, lossless methods gain smaller level of compression compared to lossy ones. Still, the lossy methods may lose some clinically significant information so they should be used in applications where certain degree of distortion is tolerable [12]. Lossy signal compression methods are divided into three groups: direct, transform and parameter extraction methods. Direct techniques, extract redundancies within the time domain signals. The advantage of these techniques is their low complexity operation [13], but its main disadvantage is that most biomedical signals are not sparse in the time domain. Transform methods typically concentrate on the energy distribution of the signal in a domain other than the time domain. Transforms such as the Discrete Cosine Transform (DCT), Discrete Wavelet Transform (DWT) and the Fourier Transform(FT) are commonly used with ECG signals. Among the transform methods, wavelet transform-based methods provide the most promising technique for ECG signal compression [14]. Examples of this technique are the work done by Benzid et al. which they compressed ECG signals by zeroing a fixed percentage of wavelet coefficients [15, 16] (SPIHT) which the authors extracted the inherent similarities across the sub-bands in a wavelet decomposition of ECG signals to compress signals. Despite the very good reconstruction quality, the main disadvantage of the wavelet transform is that its operation is computationally intensive. Parameter extraction methods only extract significant characteristics of the signal and are used for classification purposes. Examples of such methods include peak picking [17] and a long-term prediction approach [18]. Authors in [19] in order to resolve limitations of previous approaches, proposed a model that is based on Hermite and sigmoid functions combined with piecewise polynomial interpolation for exact segmentation and low-dimensional representation of individual ECG beat segments. These techniques are not suitable in many clinical scenarios. In 2006, Donoho [5] was the first to propose a compression method(transform method) called CS that transferred computational load from the sensor (encoder) to the server (decoder). This technique computes a small number of compressed samples before transmission by linear projection of a sparse or compressible signal with a random sensing matrix. ECG signals, like most biological signals, are not sparse in the time domain, so they can be made sparse like the work done by [20] and authors previous work [21] or using a deterministic or Adaptive Dictionary(AD) to sparsify the signals [22, 23]. The wavelet basis or Gaussian dictionaries are examples of deterministic sparsifying matrices. A Gaussian dictionary is based on the ECGs morphology to sparsify the signal [24]. Polania et al. [25] used wavelet transform to sparsify the frame in order to use CS, then to increase the performance of past CS techniques, they incorporated prior knowledge about wavelet dependencies across scales into the reconstruction algorithms and utilized the high fraction of common support of wavelet coefficients of consecutive ECG segments [26]. The proposed approach in [27] uses an over complete wavelet dictionary, which is then reduced by means of a training phase. Moreover, the alignment of the frames according to the position of the R-peak is proposed, such that the dictionary optimization can exploit the different scaling features of the ECG waves. The work done in [28] improves the signal sparsity through the extraction of signals significant features from each frame in order to use CS. DWT dictionaries are used in the mentioned technique. The main problem with deterministic dictionaries is their poor CS recovery quality. In [29], an iterative learning process from the test signals is used to generate a multiscale dictionary for the recovery of ECG signals. In order to increase the performance, Craven et al. [30] utilized two different patient-specific learned dictionaries for the recovery of ECG signal components: without QRS complex and with QRS complex. AD, according to signal characteristic, is employed for sparse recovery and signal reconstruction. Results show that learning dictionaries instead of using deterministic ones improves the performance and quality of the signal [31, 32]. It is worth mentioning that craven et al. used an analog-CS in their work. Despite the performance improvements made by CS techniques, they still have a persistent gap from the most beneficial transform-based compression methods such as SPIHT when energy-based distortion metrics such as PRD are considered. This motivates interest in new CS-based approaches that will improve performance in terms of signal quality. Considering the constraints mentioned above, in this work with the help of frame alignment, a redundancy removal technique is used to remove high variance between samples in the measurement vector before transmission.

## Background

This section begins by covering some important features of the theory of CS. Then, a brief introduction to ECG and dictionary learning is provided. Throughout this paper, bold lower-case letters are used to denote a vector. e.g., ***x***, and bold upper-case letters for matrices, e.g., **X**. Scalar values are indicated by italic symbols, such as *x*. Additionally, $\hat{x}$ means the estimated/recovered value of vector ***x*** and $\bar{x}$, means the average of vector samples. The list of all notations and symbols used for the following discussions of the proposed architecture are illustrated in Table 1.

**Table 1:** Definitions of some frequently used symbols in this paper.

Symbol	value	Meaning
*N*	-	Number of samples in a frame
*M*	-	Number of measurements
*B*	11	ECG bit resolution
*Y*_*mean*_	-	Mean Measurement vector
*fs*	360 HZ	ECG sampling frequency
*V*_*DD*_	0.6V	CS sampling integrator operating voltage
*FOM*	10fj	Figure-Of-Merit for Analog to Digital Converters(ADC) per conversion
*I*_*DD*_	15mA	Blackfin baseline dynamic current
*ASF*	1	Blackfin Activity Scaling Factor
*V*_*BF*_	0.8V	Blackfin operating Voltage
*AEC*	-	Average Execution Cycle in visual Digital Signal Processor(DSP)++
*CLK*	100MHZ	Blackfin core clock frequency
*B*_*T*_	-	Number of bits transmitted per frame

### Compressed Sensing(CS)

Contrary to the Shannon-Nyquist sampling theory in the Compressed Sensing(CS) framework, the number of samples taken from the signal is not determined by its maximum frequency, but by the content or information contained in the signal. Around 2004, CS pioneers Emmanuel Candès, Justin Romberg, Terence Tao, and David Donoho proved that reconstructing a sparse signal could be done by fewer samples than sampling theory requires [5, 33]. In particular, many signals are sparse, that is, they contain many coefficients close to or equal to zero, when represented in some domain [34]. CS as shown below takes a weighted linear combination of samples also called compressive measurements in a basis (**Φ**) incoherent from the basis in which the signal is known to be sparse(**Ψ**) $\Psi \in R^{n\times n}$.

$$y=\Phi x+n_{noise}$$ (1)

x=Ψα (2)

Where, $x\in R^{n}$ or $C^{n}$ is an input signal of length *n*, $y\in R^{m}$ or $C^{m}$ is the measurement vector of length *m*, $\alpha \in R^{n}$ is the sparse coefficient vector of length *n* and $\Phi \in R^{m\times n}$ or $C^{m\times n}$ (*m* < *n*) is a *m* × *n* random measurement matrix.

Incoherency between these two bases (**Ψ** *and* **Φ**) is one of the important conditions under which recovery is possible. For constructing measurement or sensing matrix, independent-identically distributed (i.i.d) entries formed by sampling a Gaussian distribution is used. With this matrix, one could be sure that its coherence with any sparsifying dictionary is small enough. The results found by [5, 33] showed that the number of these compressive measurements is proportional to the sparsity of the input signal and can be far smaller than the length of the signals and still contain nearly all the useful information. Therefore, the task of recovering the signal involves solving an underdetermined matrix equation. However, adding the constraint that the initial signal is sparse enables one to solve this underdetermined system of linear equations. To enforce the sparsity constraint when solving the underdetermined system of linear equations, one can minimize the number of non-zero components of the solution using *ℓ*_0_-norm. When measurements may contain a finite amount of noise, basis pursuit denoising algorithm is used at the destination:

$$\begin{array}{c} \text{min}\;{\left\|\alpha \right\|}_{0}\;\text{s.t}\;{\left\|y-\Phi \Psi \alpha \right\|}_{2}<\epsilon , \end{array}$$ (3)

Where *ϵ* is the bound on noise energy. The equation above can be solved by various methods like, convex relaxation, such as basis pursuit denoising and greedy algorithms such as matching pursuit and orthogonal matching pursuit [35].

### ElectroCardioGram (ECG) signal

**Fig 1:**
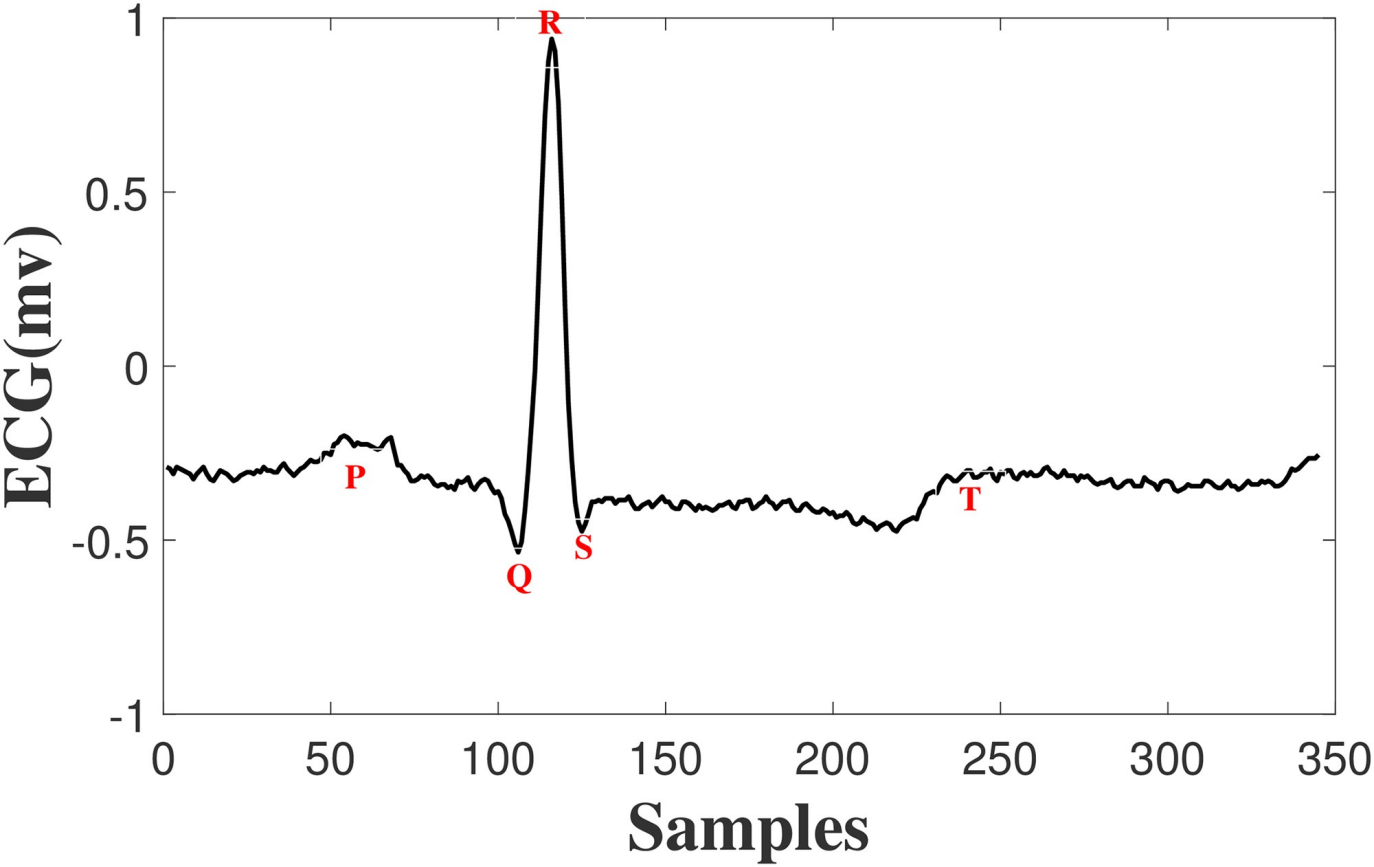
A normal ECG beat.

**Fig 2:**
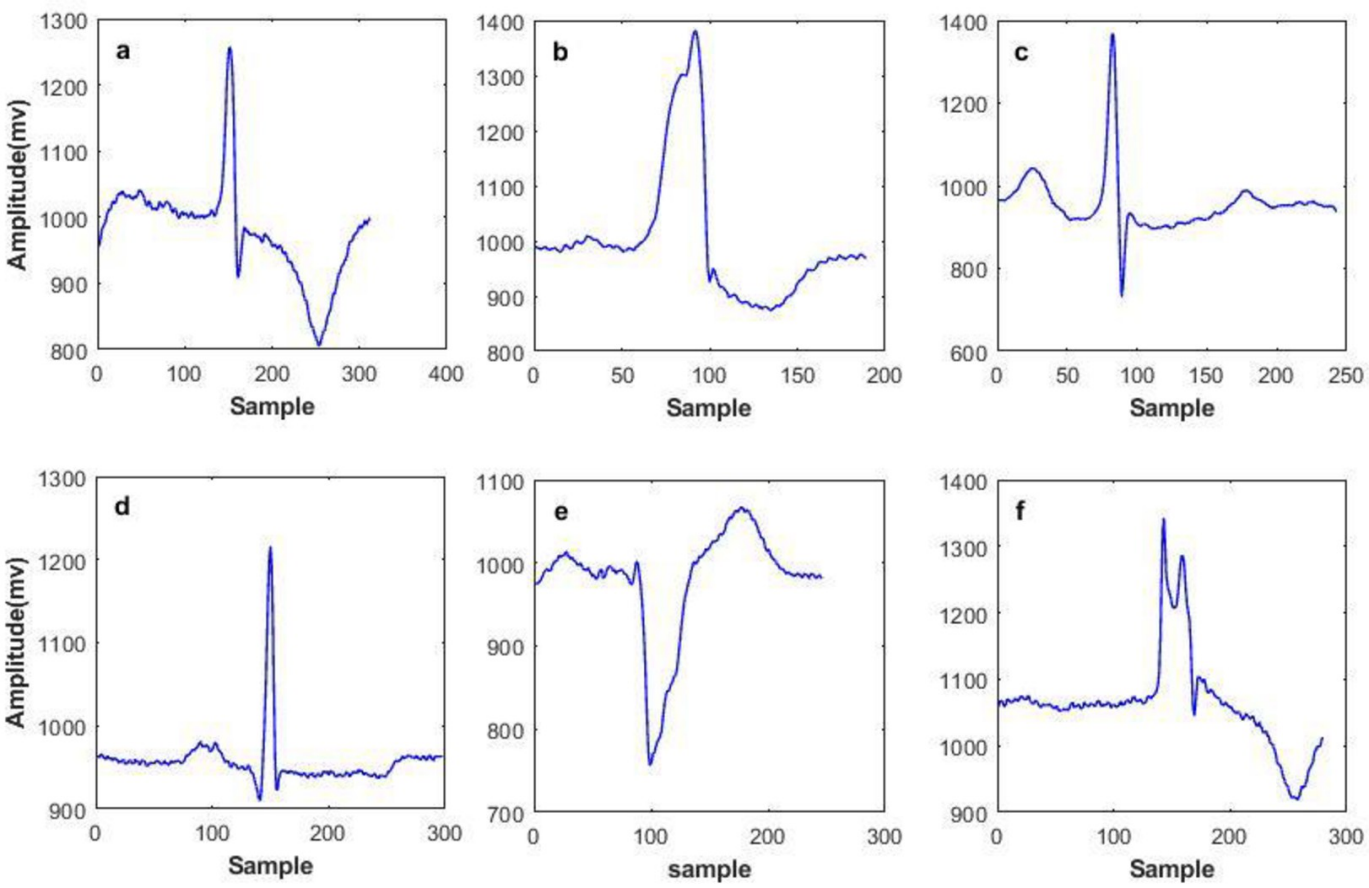
Various ECG beats (a) normal beat from record 217 (b) VEB from record 215 (c) SVE from record 220 (d) normal beat from record 100 (e) VEB from record 102 and (f) Unknown beat from record 217.

Each time the heart beats, an electrical impulse (wave) travels through the heart, causing squeezing and pumping blood from the heart. This electrical activity, which is detected by sensors attached to the skin, is called ElectroCardioGram (ECG). ECG shows two kinds of information, first the rhythm of the heartbeats (steady or irregular) and second the strength and timing of the electrical impulses as they move through different parts of the heart [36]. This signal is characterized by five peaks and valleys represented by the letters P, Q, R, S, T and Sometimes U wave is also present. In Fig 1, an ideal ECG wave is shown. As the heart experiences depolarization and repolarization, the electrical currents that are generated, spreads within the heart. The depolarization wave that spreads throughout the atria is called P and when the depolarization reaches the ventricular and spreads through them, QRS complex is produced. The T wave at the end represents the recovery of the ventricles. Changes in ECG morphology can be a sign of many cardiac arrhythmias. Although 15 various types of arrhythmias exist, AAMI classifies them into five super classes: Normal (N), Ventricular Ectopic Beat (VEB), SupraVentricular Ectopic beat (SVE), Fusion beat (F) and Unknown beat (Q) [37]. VEB is occurrence of an extra heartbeat in one of the two lower pumping chambers resulting in exclusion of p wave and unusual-shaped (wide) QRS complex that could be in multiform [38]. Atrial irritability with signs of premature beat, narrow in width but slightly different shaped than patients “normal” beats [39] is called SVE. F occurs when a supraventricular and a ventricular impulse overlap to produce a hybrid complex with an intermediate width and morphology [40]. It is worth mentioning that cardiac arrhythmias are not the only reason causing ECG morphology changes, rather different persons have their individual and unique heartbeats. A pictorial illustration of different beats from different patients and different classes are shown in Fig 2, as one could see, some ECG beats are pseudo similar.

### Dictionary Learning(DL)

As stated earlier, CS enables sub-Nyquist rate sampling of signals if the signal has a sparse illustration in a specific dictionary [5, 34, 35]. Using the standard wavelet dictionary as a sparsifying matrix offers acceptable signal quality at the receiver but fails to recover the original signal when fewer number of measurements is received. So an overcomplete dictionary of signal components known as atoms, built from a set of training signals has shown to provide a significant performance improvement [41]. Dictionary learning (DL) is an iterative learning which uses a set of *t* training signals $X_{T}=\left[x_{1},x_{2},x_{3}\ldots x_{t}\right]\in R^{N\times t}$. The purpose of this process is to iteratively improve the dictionary $\Psi \in R^{N\times p}$ by reaching sparser representations of the training signals and revising the dictionary based on the current sparse representations $\beta \in R^{p\times t}$. For constructing the dictionary, an optimization problem must be solved:

$$\underset{\Psi ,\beta }{\text{min}}\;\left\{{\left\|X_{N,t}-\Psi_{N,p}\beta_{p,t}\right\|}_{F}\right\}$$

$$\;\text{subject}\;\text{to}\;{\left\|\beta_{i}\right\|}_{0}<S\left(i=1,...,t\right),$$

Where *ℓ*_*F*_-norm is the Frobenius norm and the process is bound by a sparsity constraint, such that the sparsity level *S* is the maximum number of non-zero entries in each sparse representation ***β***. Aharon et al. [42] proposed a DL algorithm called K-SVD, which is used in this paper.

## Proposed scheme

**Fig 3:**
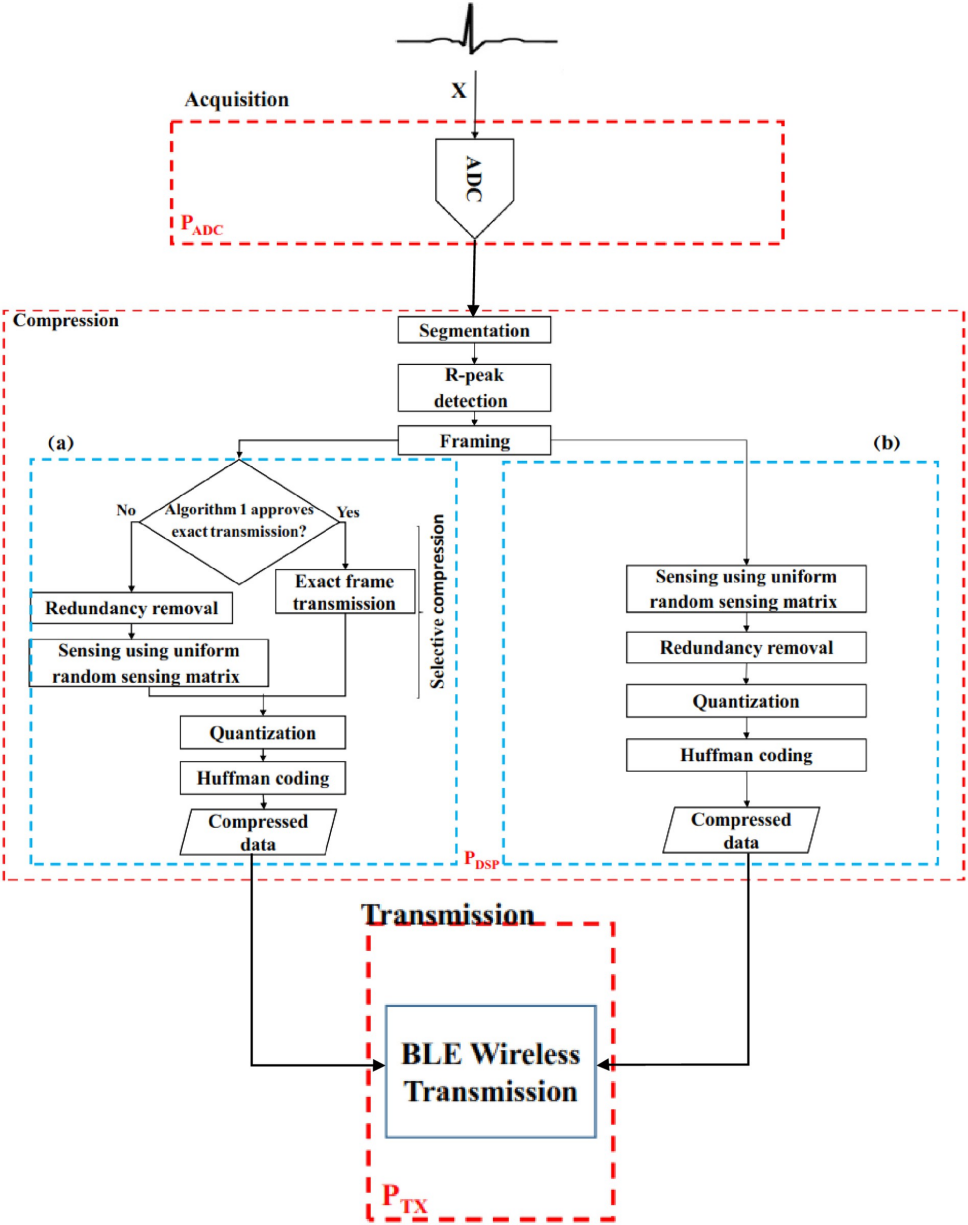
The workflow of the a) scheme in [21], b)proposed scheme.

The objective of this study is to enhance energy efficiency while preserving the performance of the recovered signal in terms of signal quality. So in order to improve performance, the redundancy could be removed from two stages, either before CS like the authors’ previous work [21](Fig 3a) or after CS. The workflow of the proposed scheme is reviewed in Fig 3b. The workflow of the proposed technique segmentize the digitized signal into 512 samples, enough to contain at least one cardiac cycle. By detecting R-peaks on every segment, a frame of aligned R-peak with previous R-peaks is constructed. In this stage, despite the work done in [21] compressed sensing is applied directly to the aligned frames, resulting in high redundant measurement vectors. By removing the mean measurement vector from the measurements, samples clustered near 0 are produced, ensuring reduced bits per sample for quantization. The workflow consists of the following stages. A description of each step is presented in detail in the following subsections.

1. Segmentation
2. R-peak detection
3. Heartbeat Framing
4. Redundancy removal of compressed measurements
5. Quantization and Huffman coding
6. Signal reconstruction

### Segmentation

**Fig 4:**
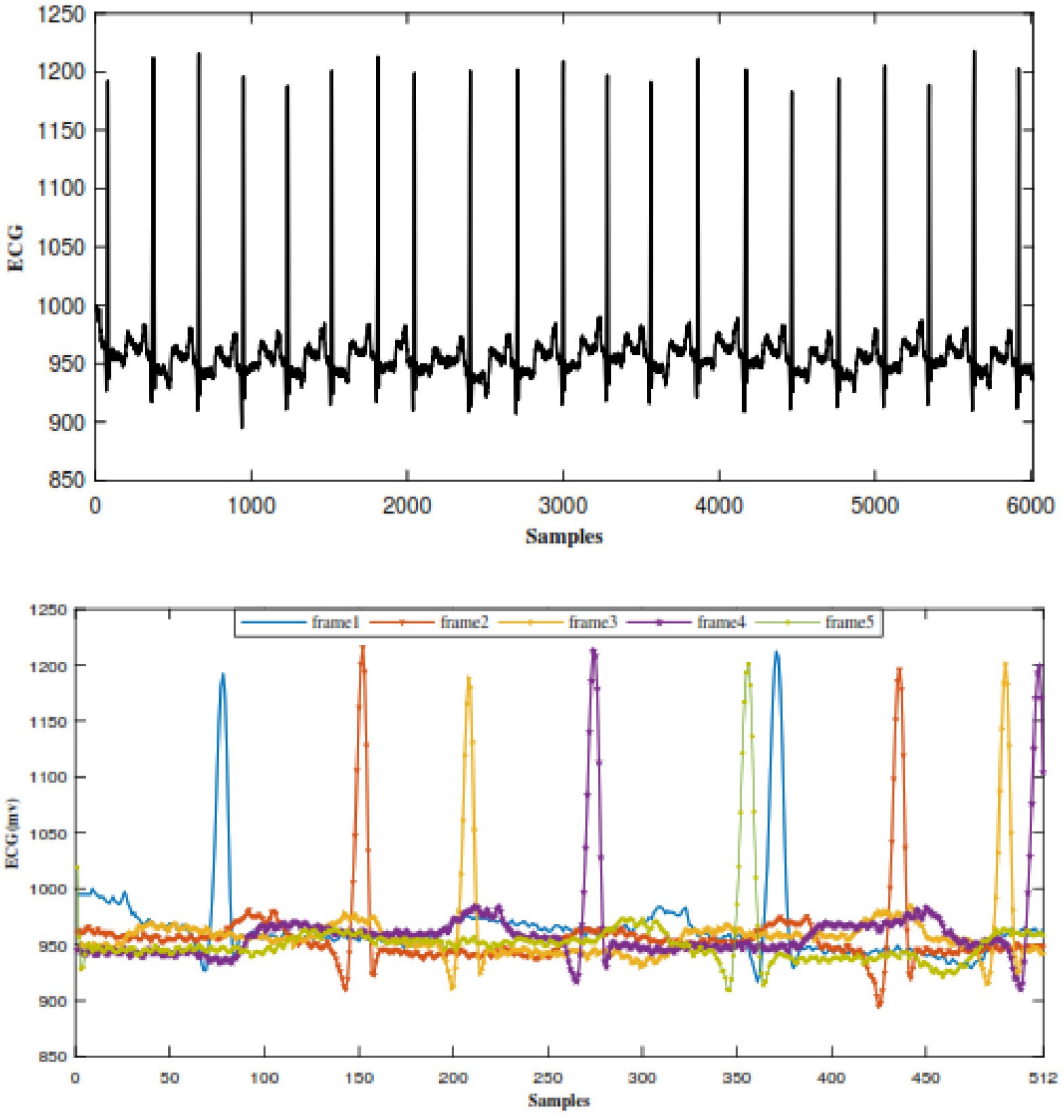
ECG signal. (a) Continuous ECG signal. (b) Consecutive ECG frames.

Fig 4a shows a continuous ECG signal from the MIT-BIH arrhythmia database. In order to be able to detect beats correctly and to keep the acquisition time sufficiently short for real-time monitoring, each segment should contain at least one beat. So as shown in Fig 4b, a 512-sample segment is defined as an observation window equal to approximately 1.5s at 360 HZ. This segment should in general cover important features(P-QRS-T) of at least one beat.

### R-peak detection

A complete cardiac cycle detected by lead II consists of a local maxima(R-peak) which changes significantly compared to the other peaks and valleys of the cycle [43]. Previous work done by the authors contends an energy-efficient peak detector that used the slope and amplitude difference along with the peak interval of candidate samples to detect R-peaks [21].

For performance evaluation of the proposed peak detection, the information of true R-peaks is collected from the MIT-BIH database. In Table 2 the performance of the QRS detection using the proposed technique for various records is compared with the records annotation. The performance metrics shown in Table 2 are the True Positive (TP)-which shows the true detected beats—and False Negative(FN)- which shows undetected true beats. Sensitivity (Se), which measures the percentage of true positives among the identified and unidentified QRS complexes: *Se*(%) = (*TP* ÷ (*TP* + *FN*)) × 100(%).

**Table 2:** Performance of QRS detection technique.

ECG data	TP	FN	Sensitivity (SE)
**100**	2273	0	100%
**101**	1864	1	99.94%
**202**	2115	2	99.90%
**230**	2256	0	100%
**Additive**	**all**	3	99.96%

As one could see, the ability of the proposed technique to correctly detect annotated R-peaks is higher than 99.95%.

### Heartbeat framing

**Fig 5:**
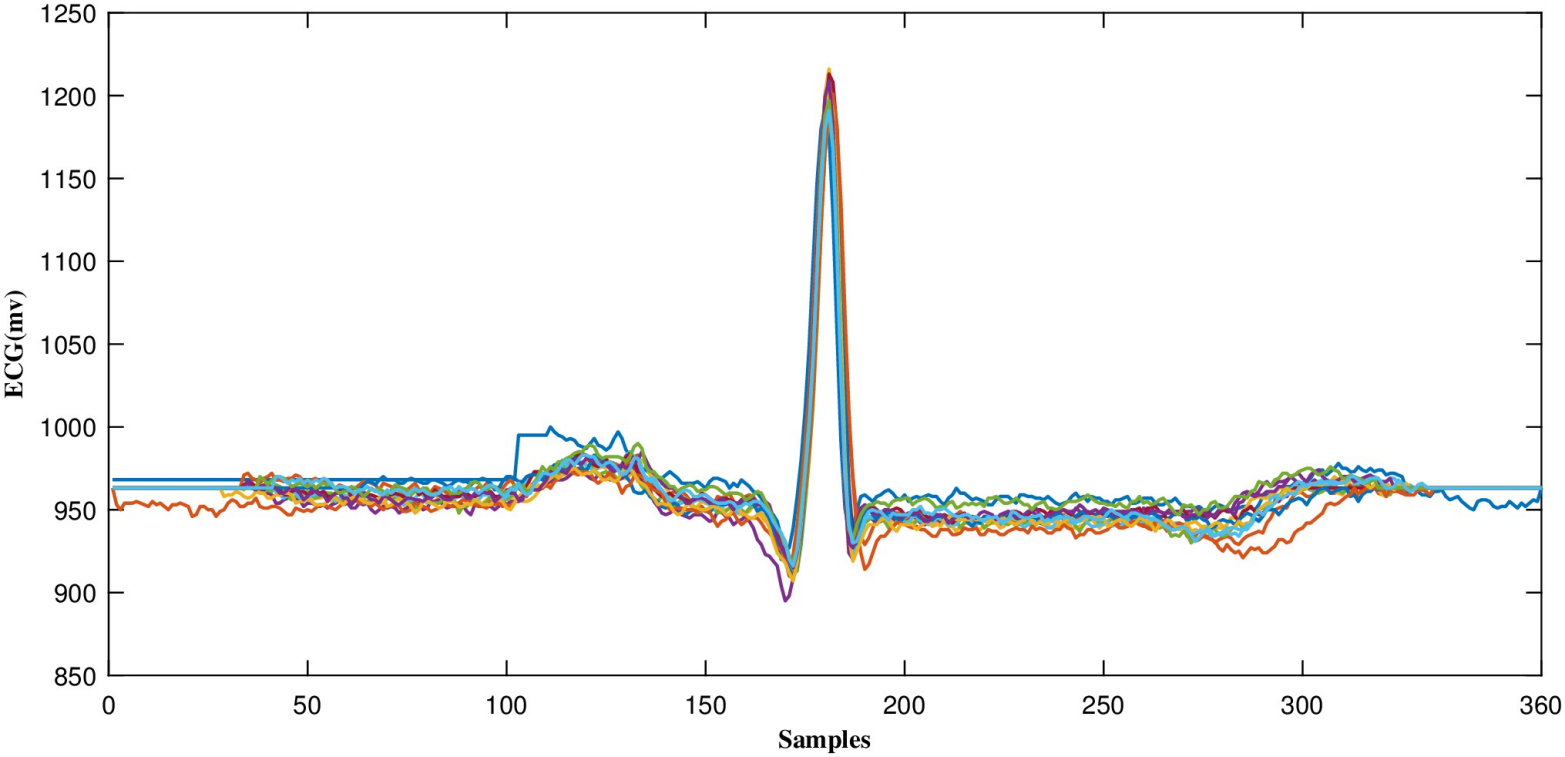
An example of aligned ECG segments.

Frames of equal duration are created from detected R-peaks in the midpoint, covering other parts of a complete heartbeat, as can be seen in Fig 5. The technique used to align R-peaks is relevant to the method defined in [44]. In the mentioned technique, in order to locate a complete heartbeat, three consecutive R peaks are used. To be able to further process a frame with a complete heartbeat, its duration has to be fixed. According to the previous work done by authors [21], this value(D) is set to 360. The process of heartbeat alignment is vital to the high performance of the proposed technique. Since CS measurement vectors of aligned heartbeats are highly correlated and similar, without the R-peak alignment, the R peaks could be in any possible locations, causing variability in the measurement vectors.

### Redundancy removal of compressed measurements

As shown in Fig 2, ECG frames seem highly similar in most of the classes except when a beat represents a VEB. In order to prove this impression, Table 3 represents the correlation between different classes shown in Fig 2. It can be seen that one kind of VEB is less correlated to the other classes, compared to other pairs.

**Table 3:** Correlation matrix.

Normal beat(217)	100%				
**VEB**	60%	100%			
**SVE**	44%	57%	100%		
**Normal beat(100)**	79%	59%	81%	100%	
**VEB (PVC)**	−68%	−62%	−46%	−66%	100%
	**Normal beat(217)**	**VEB**	**SVE**	**Normal beat(100)**	**VEB (PVC)**

In one form of VEB, known as Premature Ventricular Contraction (PVC), the QRS complex has greater width and higher-than-normal amplitude and is usually opposite in polarity to a normal QRS complex [45]. Hence, before applying a random sensing matrix to a frame, the existence of the mentioned beat type should be verified by comparing its peak amplitude with the mean value of the frame. If the peak amplitude is less than the mean value, the existence of a PVC is detected. There are several dedicated methods in the literature for frame classification [46–49]. Though these methods accurately classify ECG frames, their processes require intensive mathematical operations.

For the performance evaluation of this approach, it was verified if a QRS complex is genuinely a PVC or not. The annotations of the QRS complexes were collected from the MIT-BIH database and were compared with the proposed technique. In Table 4, the performance of the proposed PVC detection procedure is shown in terms of the misclassification rate.

**Table 4:** Percentage of misclassification.

ECG data	Misclassification Rate(%)
**100**	0%
**106**	5%
**107**	10%
**109**	9%
**114**	2%
**124**	6%
**205**	0%
**Average**	4.5%

However, as the operation of this algorithm depends on a simple comparison, for noisy environments, this method doesn’t outperform state-of-the-art techniques. So by considering the trade-off between the energy consumption and performance, this algorithm was used. After beat identification, a measurement vector was produced by applying a Gaussian random sensing matrix to the frame.

**Fig 6:**
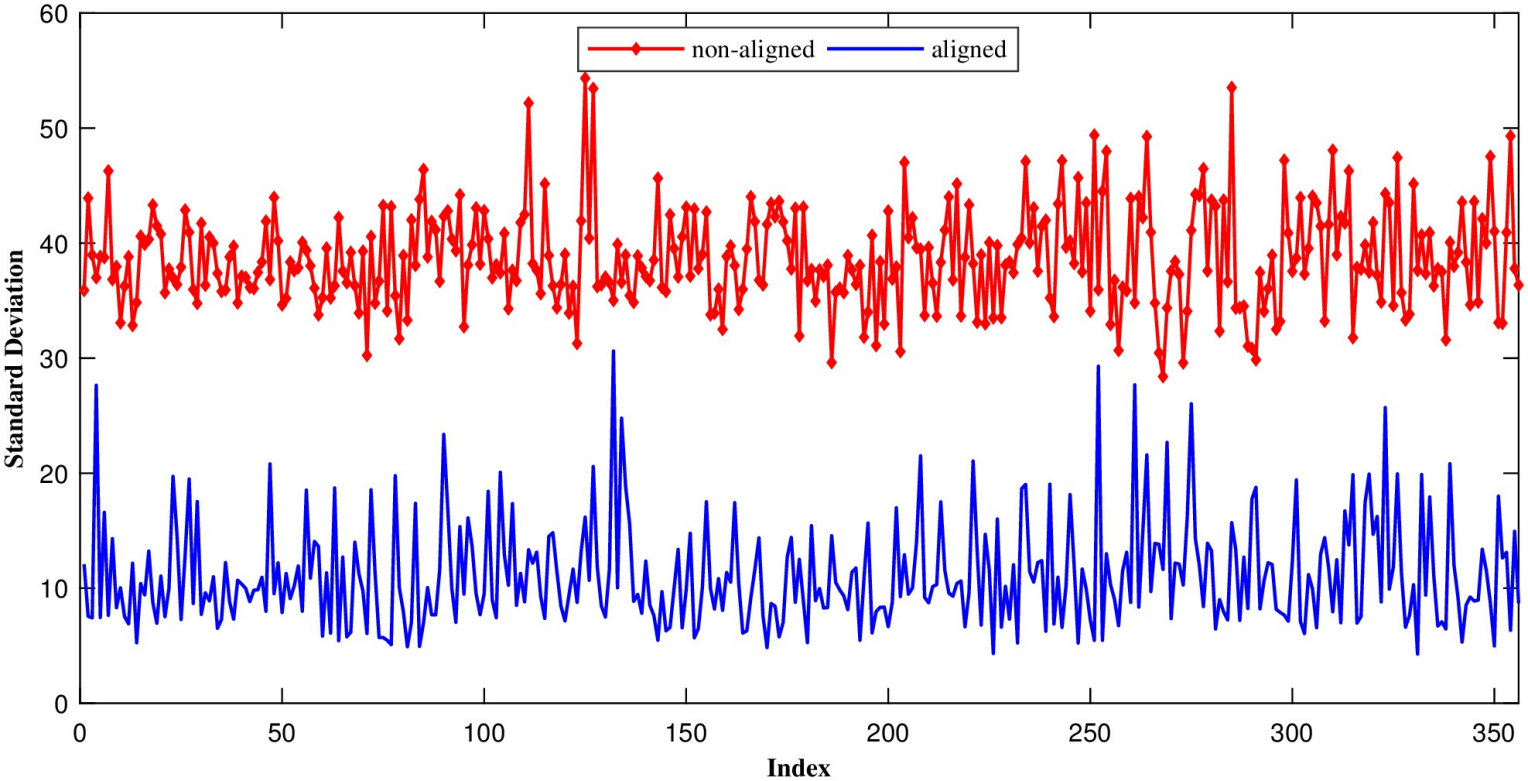
Standard deviation(around each entry) of the measurement samples over multiple consecutive measurement vectors.

As mentioned earlier, because ECG beats are pseudo-periodic and R-peaks are aligned, there exists a high redundancy in measurement vectors. Fig 6 plots the samples standard deviation (around each entry) of multiple consecutive measurement vectors that resulted from applying CS to aligned and non-aligned ECG frames of record number 100. As can be seen, the standard deviation of each entry is lower when ECG segments are aligned, indicating the significant amount of redundancy existent in the data.

Hence, a redundancy removal method using a mean measurement vector, computed off-line and trained using 90% of measurement vectors [22], was applied to remove the redundancy from the measurement vectors. Here, according to the presence of a PVC beat in the ECG frame, the corresponding mean measurement vector $\bar{Y}$ was subtracted from the current measurement vector and the difference vector was processed. The mean measurement vectors are generated as follow:

$$\begin{array}{c} y_{i}\left(PVC\right)=\frac{\sum_{j=1}^{T}Y{\left(PVC\right)}_{t<i,j>}}{T} \end{array}$$ (4)

$$\begin{array}{c} y_{i}\left(NPVC\right)=\frac{\sum_{j=1}^{T}Y{\left(NPVC\right)}_{t<i,j>}}{T} \end{array}$$ (5)

Here $\bar{y}\left(PVC\right)$ and $\bar{y}\left(NPVC\right)$ are the mean measurement vectors of M entries, corresponding to ECG frames with PVC beat and other pseudo similar beats, respectively. ***y***_***t***_ is the training measurement vectors, T is the number of measurement vectors used in training, i indexes through each vector and j indexes through all training frames from 1 to T. This mean measurement vectors are created off-line and is existent at both source and destination.

**Fig 7:**
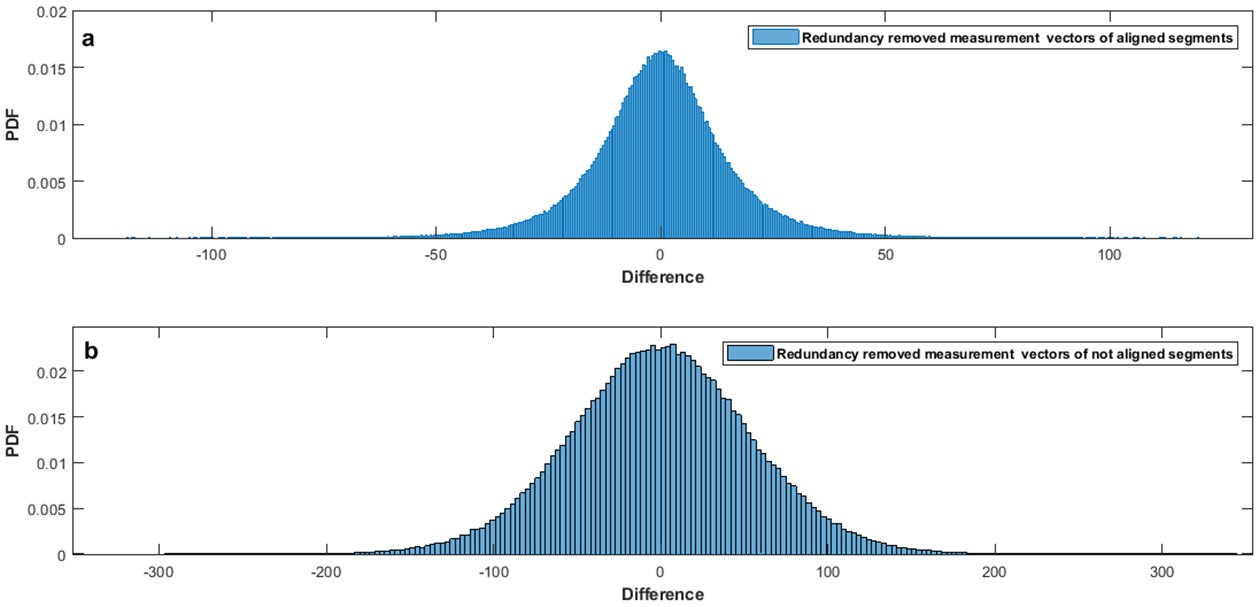
PDF curves of difference values of redundancy removed a)aligned and b)not-aligned frames.

Removing the corresponding redundancy from the remaining 10% of measurement vectors, eventuates in samples to be clustered near 0 as shown in Fig 7a. It is concluded from Fig 7 that when ECG frames are aligned, the chance of using lower than 9 bits for representing each sample increases.

### Quantization and Huffman coding

Without redundancy removal, 11 bit is desirable to represent compressed sensed records from the MIT-BIH database. Mamaghanian et al. managed to reduce this amount to 9 bits via removing static redundancy [22]. To reach an 8-bit quantizer, the LloydMax algorithm, which is a non-uniform quantization technique, is used by polania et al. [26]. The proposed method will tackle the previous works by aligning frames, therefore, it would be able to reach smaller quantizer. It is evident from Fig 7a that the distribution of redundancy removed measurements has more mass near zero. So in this situation, choosing smaller quantization intervals in high mass regions and larger intervals away from the origin would be a right choice [50]. This kind of quantizers are called non-uniform quantizers because of non-uniform intervals. Step sizes for high and low mass regions are chosen according to the technique defined in [51].

$$\Delta_{h}\approx \frac{L}{\left(2^{q}\right)\times \left(0.7\right)}$$

$$\Delta_{l}\approx \frac{L}{\left(2^{q}\right)\times \left(0.3\right)}$$

Craven et al. choose 0.7 and 0.3 as the optimal ratio between high and low resolution reigns. *L* is the limit measurement value and *q* is the bit resolution, which in the proposed technique is 5. The quantizer and Huffman dictionary are created off-line from the training data related to the non-uniform nature of the redundancy removed measurements [50].

**Fig 8:**
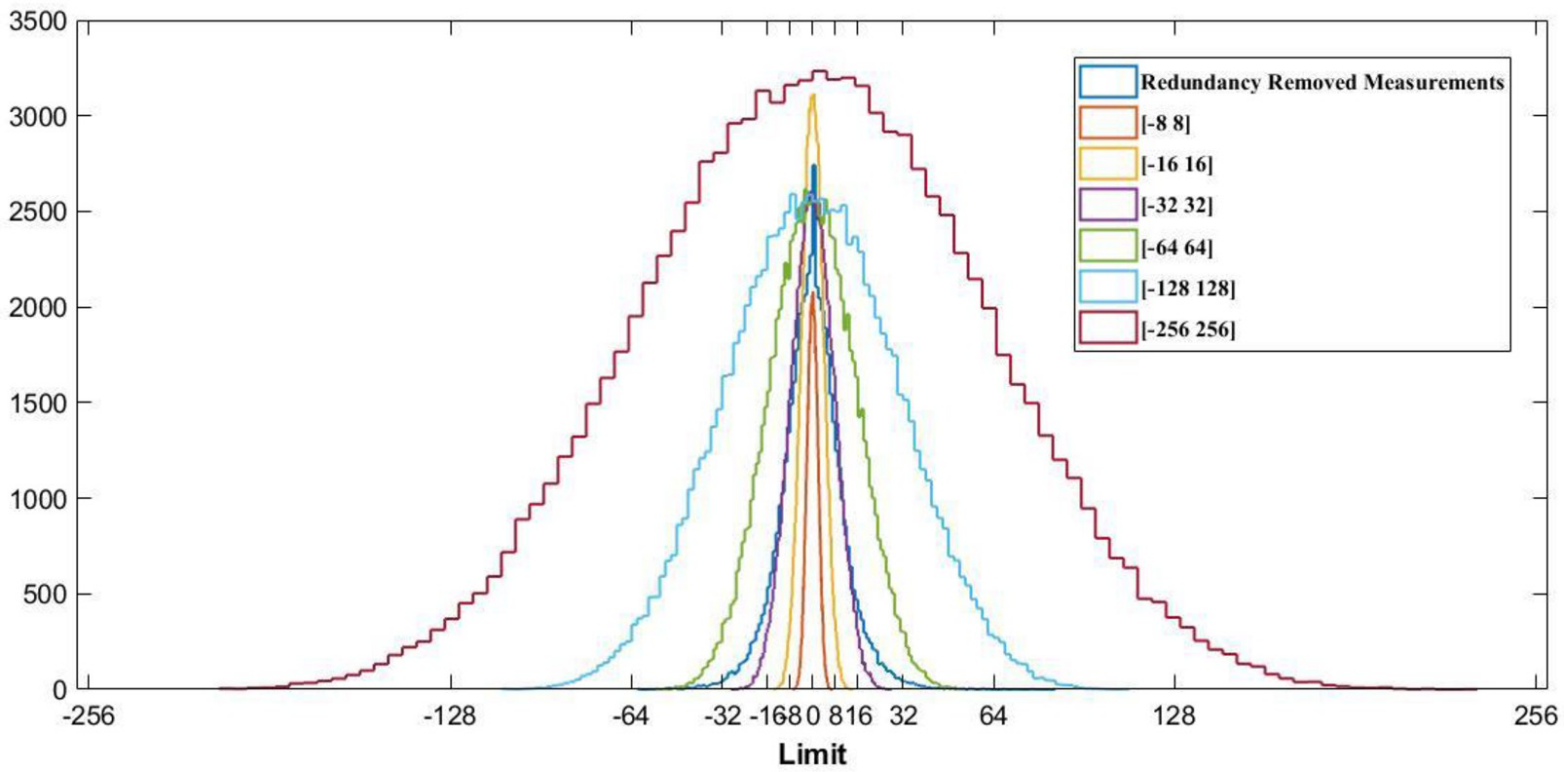
Different non-uniform distributions.

In order to choose an optimal value for *L* to cover the redundancy removed measurements and to reach minimum granular and overload quantization error, analyses have been done on training signals. As shown in Fig 8, the ranges that cover the redundancy removed measurement vectors entirely, are from [-64, 64] to [−256, 256]. To choose the optimal limit, different scenarios are tested, and according to them [-64, 64] is the optimal range that can satisfy the mentioned goals.

### Signal reconstruction

The received signal in this stage consists of 2 fragments. First comes the Huffman coded data and then a bit indicating the presence of PVC in the sent frame. As noted earlier, Huffman dictionary, mean measurement vectors, sparsifying dictionaries and sensing matrix are created offline and are existent at the destination. After decoding the received Huffman coded data, the corresponding mean measurement vector is added to the decoded frame. Then for recovery of each frame type (PVC beats and non-PVC beats), basis pursuit denoising algorithm and corresponding patient-specific dictionary is used.

Though the employment of patient-specific dictionaries requires adequate training signals from an individual patient, as shown in Fig 2a and 2d each patient may have unique ECG morphology for each beat type. So training a dictionary with patients’ own ECG signals improves the recovered signal quality compared to situations where patient-agnostic dictionaries are used.

## Simulation and results

All the ECG records from the MIT-BIH arrhythmia database (MITDB) containing normal and abnormal beats with time-varying QRS morphology were used [52]. The database includes 48, half an hour ECG records, digitized with a sampling rate of 360 HZ with an 11-bit resolution. In order to compare the proposed technique with the existing literature, the modified limb lead II channel is used.

### Performance evaluation metrics

To quantify the performance of the compression algorithms, Compression Ratio(CR) metric is used. An energy-based distortion metric known as PRD is used for evaluating the quality of the reconstructed signal. Sensitivity (Se) and SPecificity (SP) of QRS detection is also used. The proposed method was evaluated based on the recommendation of the American National Standard for ambulatory ECG analyzers (ANSI/AAMI EC38–1994) [37].

- Compression Ratio (CR) is a measure of the reduction in the number of bits needed to represent the original signal:
  $$\text{CR}≜\frac{N\cdot B_{o}}{B_{T}},$$
  where *N* is the number of samples of the original signal with *B*_*o*_-bit resolution and *B*_*T*_ is the number of transmitted bits.
- Percent Root-mean-square Difference (PRD) measures the quality of the reconstructed signal:
  $$\text{PRD}\;\left(\%\right)≜\sqrt{\frac{\sum_{n}{\left(x\left(n\right)-\hat{x}\left(n\right)\right)}^{2}}{\sum_{n}{\left(x\left(n\right)-\bar{x}\right)}^{2}}}\times 100\%.$$
- Sensitivity (Se), which measures the percentage of true positives among the identified and unidentified QRS complexes:
  $$\text{Se}\;\left(\%\right)≜\frac{TP}{TP+FN}\times 100\%.$$
- Specificity (SP), which measures the ability to correctly identify non-QRS complexes:
  $$\text{SP}\;\left(\%\right)≜\frac{TN}{TN+FP}\times 100\%.$$

### The compared algorithms

With the help of different state-of-the-art methods, we demonstrated the performance improvement of the proposed technique. These methods include the work done in [27, 31, 32], which we used to compare the acquired PRD, energy consumption, Se and SP.

### Experimental results and discussion

In this section, the experimental setup used to qualify the performance of the implemented technique is introduced first, then validation of the proposed method using a series of experiments is started. The quality of the reconstructed signal is compared with the state-of-the-art techniques. In the end, the power profile of the sensor is shown for different CRs.

#### Experimental setup

Choosing an appropriate sensing matrix in order to satisfy the key restricted isometry property (RIP), selecting proper sparsifying dictionary (***ψ***) size, building suitable quantizer and associated Huffman dictionary and train/testing partition sizes for calculating both mean measurement vectors and ***ψ*** affects the PRD. In order to make the coherence between the sparsifying dictionary and sensing matrix low enough, random values with Gaussian distribution are selected for the sensing matrix. Usually, for all learning processes, 90% of the data is used for training and the rest of the data is used for testing. In the proposed work, a patient-specific sparsifying dictionary is used for each patient, denoting that 90% of the past saved ECG frames of the patient are used for training the dictionary and calculating the mean measurement vectors. Hopefully, different training partition sizes have a shallow effect on PRD, indicating that the DL method can still work in scenarios where less training data is available [31].

As indicated earlier, for defining high and low resolution regions in the utilized non-uniform quantizer, a limit value is used. This value will determine the maximum value for the quantizer, and to simplify implementation, the same value is used for both positive and negative limits. initial offline testing, in the training phase on the suitable limiting values and corresponding bit resolution, shows that the optimal value for limit is 64 with *q* = 5.

#### Signal quality assessment

**Fig 9:**
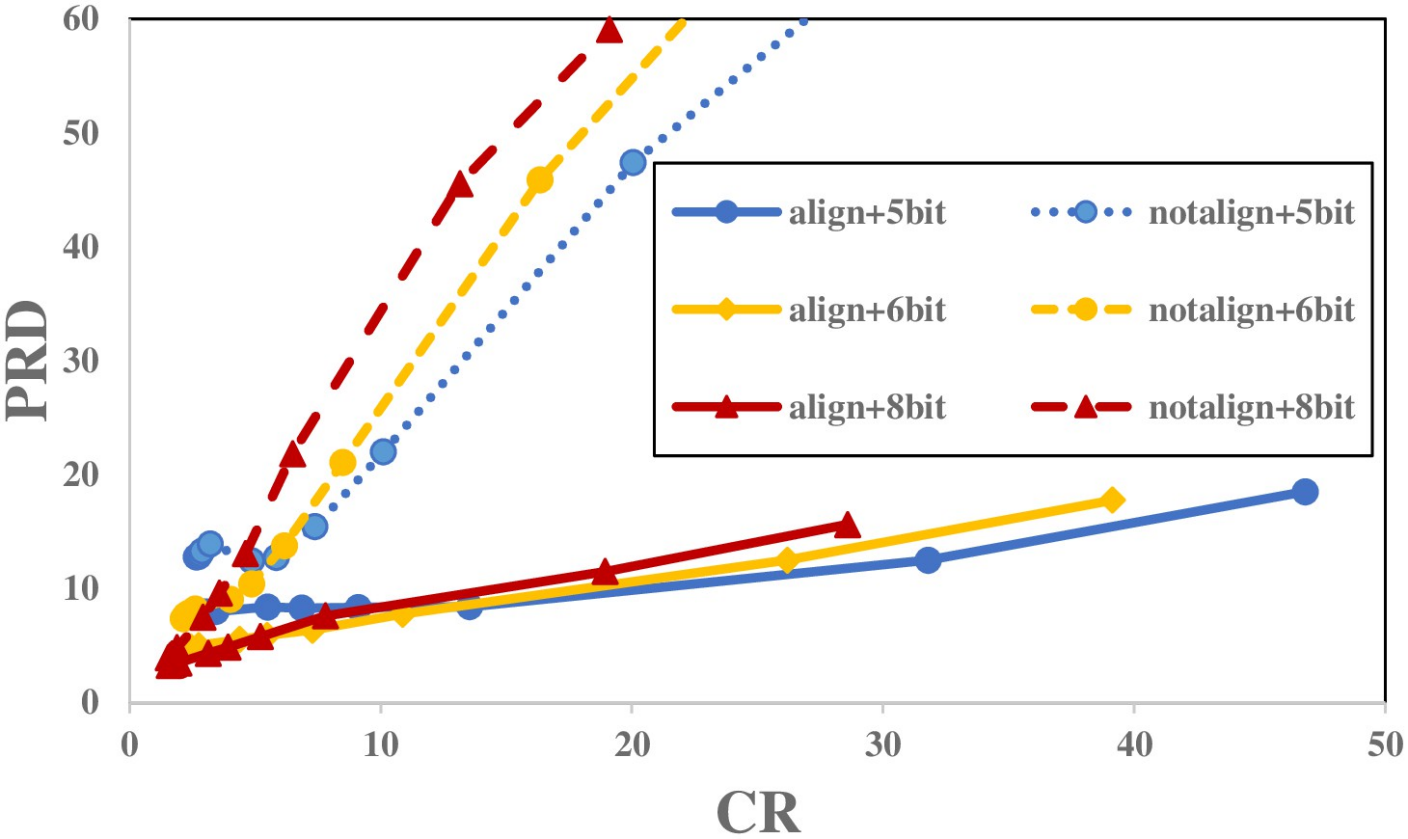
Comparison of PRD for different scenarios.

**Fig 10:**
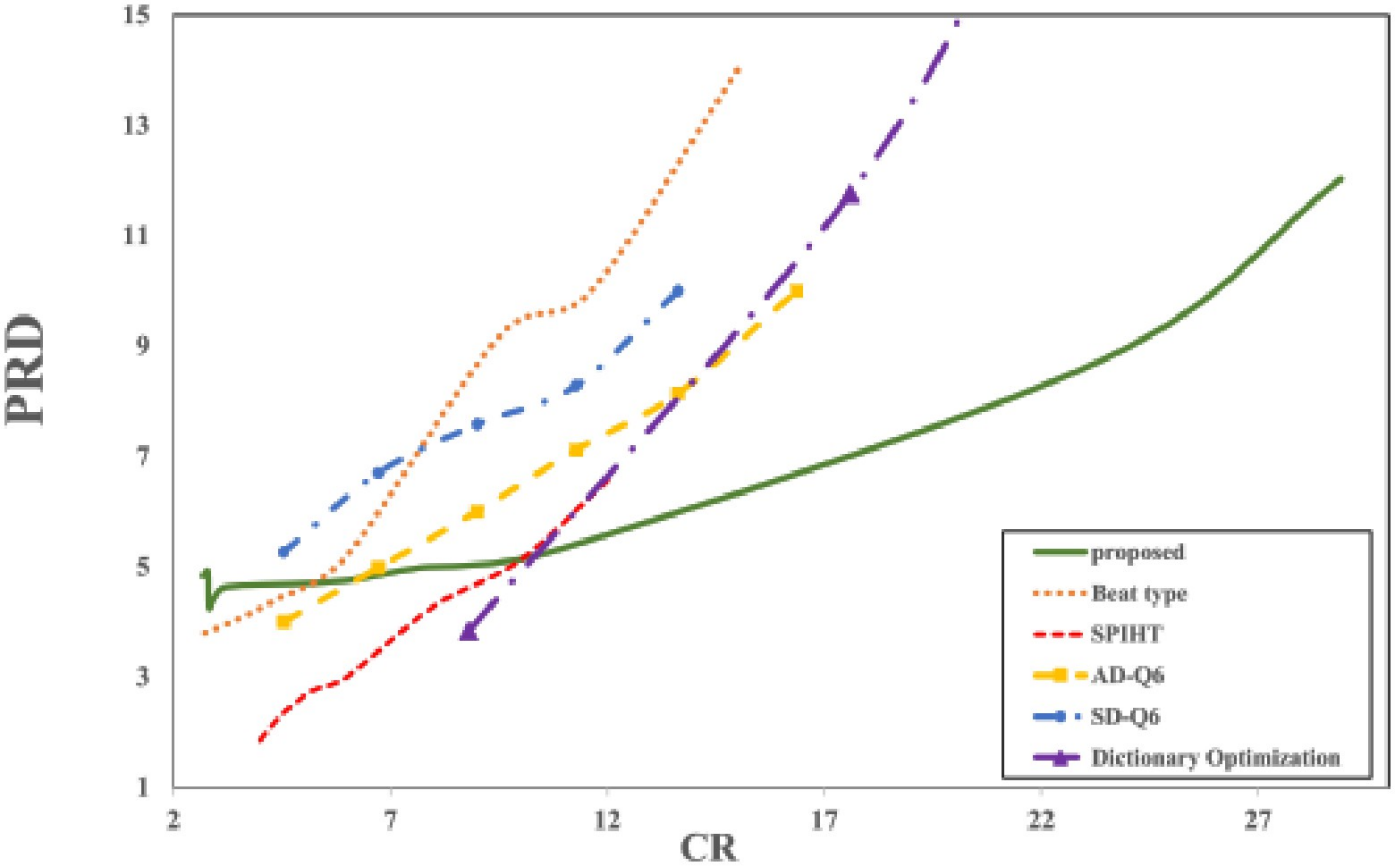
Comparison of PRD for different compression ratios.

Since ECG signals carry multiple important clinical features, the reconstructed signal has to preserve these information. PRD, as shown in Fig 9 is used for assessing the performance in terms of recovered signal quality. In order to compare the proposed technique with the state-of-the-art techniques, the MIT-BIH Arrhythmia Database is used. As previously stated, after aligning ECG frames, the calculated measurement vectors become highly similar. So reducing the corresponding mean measurement vector lowers the data range in a way that smaller quantizer related to the work done in [31] can be used. Fig 9, shows that how aligning the ECG frames can improve the performance in terms of PRD in all the scenarios for all CRs. By focusing on using 5-bit non-uniform quantizer, Fig 10 proves that by aligning ECG frames, more compression could be gained for *PRD* > 5% compared to [32] (beat_type) where beat type dictionaries are trained with not aligned frames. When ECG frames are not aligned, the QRS complexes could be in any possible location, making the sparsifying dictionaries hard to cope with and in most of the cases, leading to poor signal recovery especially for higher CRs. For example, in order to reach *PRD* = 9%, Craven et al. [31] reach a *CR* = 11.28% and *CR* = 13.68% when standard and AD respectively are used. While by aligning ECG frames, the proposed technique can reach higher CR equal to 24. Compared to SPIHT, the proposed technique only has a higher quality for *CRs* > 10 but managed to reduce the gap from that technique. By comparing the proposed technique with the work done in [27](Dictionary Optimization) which aligns the frames just for dictionary optimization, one could realize that redundancy removal on the quantizer has an striking effect on compressing the ECG frames and acquiring an optimal PRD value.

To show how well the proposed technique can preserve relevant signal characteristics, a well-known detection algorithm called Pan-Tompkins algorithm is used for the reconstructed signals. Although the primary focus of this experiments was the PRD metric, Sensitivity (Se) and Specificity (SP) are also included to indicate the accuracy of QRS detection of each algorithm as the CR is increased. In Table 5 the proposed technique is compared with the analysis done by Craven et al. [31] and the state-of-art technique SPIHT. Craven used adaptive dictionary in order to sparsify frames with QRS complexes. In his analysis he compared two different dictionaries: patient-specific and patient-agnostic. Our proposed technique provides superior performance to the work done by Craven and SPIHT across the tested metrics for different compression ratios and can maintain a high rate of QRS preservation.

**Table 5:** Comparing the ability for QRS-detection.

CR	Proposed	SPIHT	[31]
3	*Se* = 100%,*SP* = 100%	*Se* = 97.9%,*SP* = 99.8%	*Se* = 99.3%,*SP* = 99.8%
7	*Se* = 99.8%,*SP* = 100%	*Se* = 98%,*SP* = 99.8%	*Se* = 98.9%,*SP* = 99.5%
12	*Se* = 99.6%,*SP* = 99.8%	*Se* = 97.6%,*SP* = 99.8%	*Se* = 98.7%,*SP* = 99.3%

#### Evaluation of energy consumption

The proposed architecture assumed in this paper is detailed in Fig 3. In this section, the energy consumed for i)acquisition, ii) performing digital signal compression on a Blackfin (BF537) DSP [53] as an example platform, and iii)wireless transmission of the ECG frame using the Texas Instruments CC2540 BLE wireless transceiver [54] is considered. The variables used in the power analysis are defined in Table 1. Different operating characteristics associated with the Blackfin BF537 DSP were derived from [55]. Analog Devices Visual DSP++ code execution profiling tool for BF537 has been used to generate the firmware binary code and calculate the number of clock cycles.

**Fig 11:**
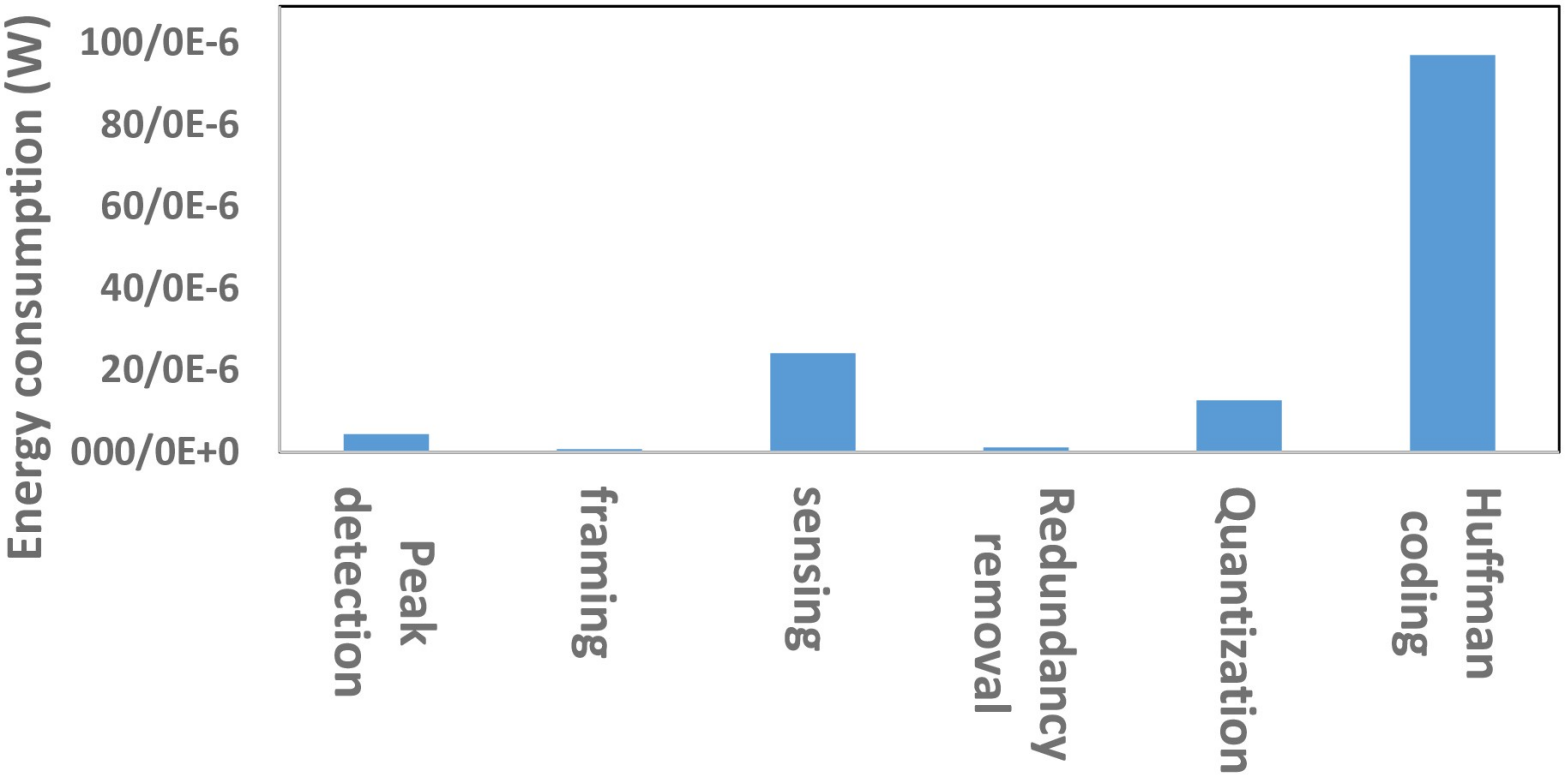
Breakdown of the energy consumption in processing part.

**Fig 12:**
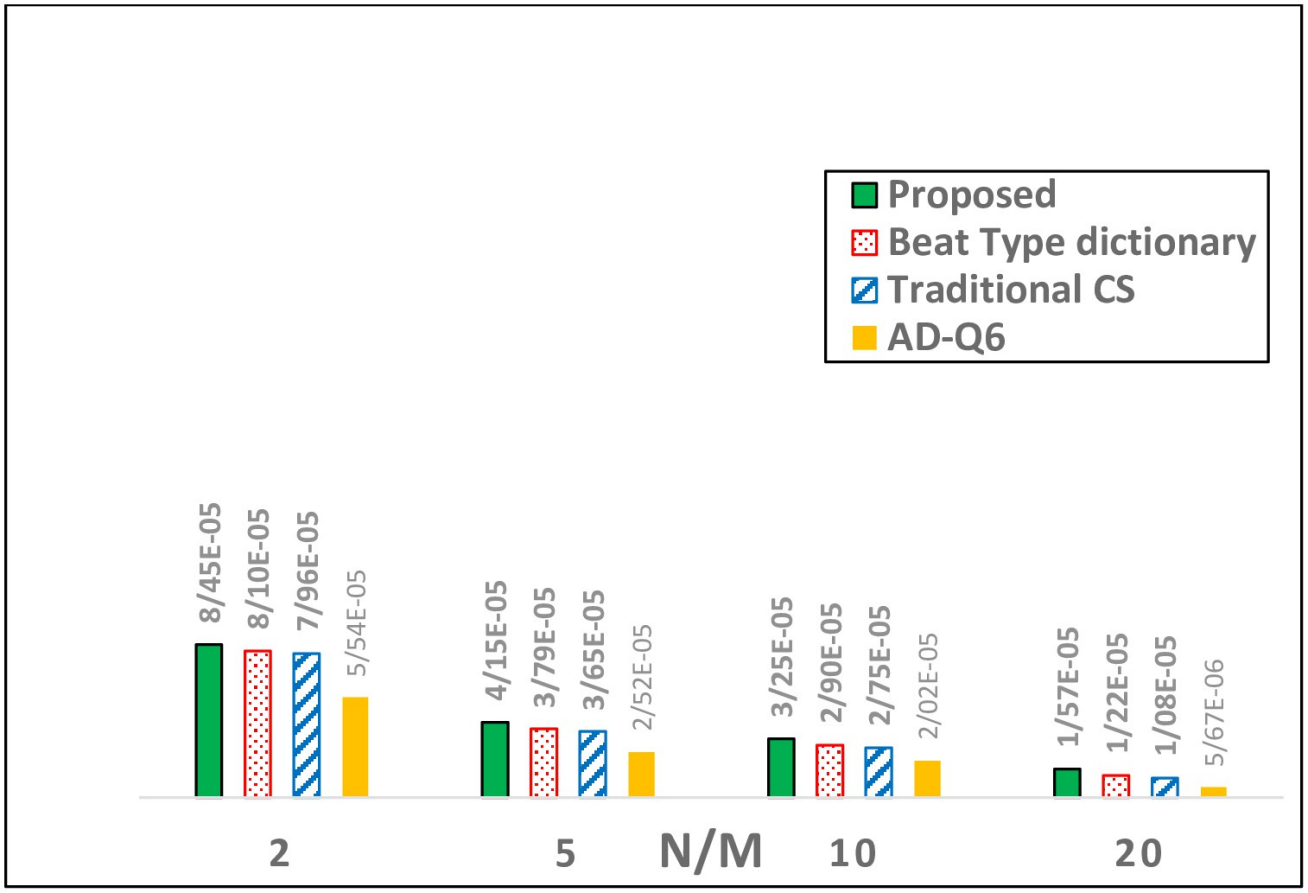
Processing power consumption (*P*_*DSP*_).

1. Acquisition
  The ADC power for the proposed technique is expressed as below. According to [56] successive approximation register ADCs are the most energy-efficient ADCs, which can provide a FOM of <10*fj* per conversion.
  $$\begin{array}{c} P_{ADC}=N\cdot 2^{B}\cdot FOM\cdot F_{s}. \end{array}$$ (6)
2. Digital signal compression
  The execution power consumption is evaluated by specifying the average number of clock cycles consumed to compress 1s of the ECG frame. The number of clock cycles (AEC) was calculated using the Analog Devices Visual DSP++ code execution profiling tool. The power required (*P*_*DSP*_) to process 1s of ECG frame was calculated as follows [55],
  $$\begin{array}{c} P_{DSP}=I_{DD}\cdot ASF\cdot V_{BF}\cdot \frac{AEC}{CLK}\cdot \frac{F_{s}}{N}. \end{array}$$ (7)
  As described before, the proposed technique detects peaks, then it aligns frames according to the detected peaks, resulting in consuming more energy in the processing part compared to the work done in [31, 32]. Table 6 shows the average clock cycle of each operation at varying CRs. As one could see, the average execution cycle and obviously energy consumption of peak detection and framing stages are not proportional to CR.
  To understand the effect of the added stages on the total energy consumption, the energy consumption breakdown of the processing part was further characterized. In particular, Fig 11 depicts the breakdown of the total energy consumption between the six main processes. As shown in Fig 11, the proposed technique does not seem expensive in terms of energy consumption in the peak detection and framing stages.
  Because of the new stages added to the traditional CS, the proposed technique has about 4.96*μW* higher energy consumption in the processing part. In Fig 12, this difference in energy consumption is shown graphically. Compared to the traditional CS and the work done in [32], the proposed work consumes 4.96*μW* and 3.54*μW* more energy in the processing part, respectively. So far, AD-Q6 has the less energy consumed because of the analog-CS used in their technique [31]. This shows that the extra stages of the proposed technique compared to plain CS are not so energy-consuming.
3. Wireless transmission
  The power consumption of Texas Instruments CC2540 BLE transceiver is 84*mW* during wireless transmission of one byte of data for the period of 8*μs*. Therefore, the required energy for transmitting one bit of data with the assumption that transceiver is turned off between transmissions is 84*nJ*. Consequently, the power consumed per transmission (*P*_*TX*_) is dependent on the level of compression, upcoming equation shows the calculation:
  $$\begin{array}{c} P_{TX}=\left(84nj\right)\cdot B_{T}\cdot \frac{F_{s}}{N}. \end{array}$$ (8)

**Table 6:** Average Clock Cycle count of each stage.

N/M	2	5	10	20
Peak detection	35980	35980	35980	35980
Framing	5375	5375	5375	5375
Sensing	201567	94212	60765	42458
Redundancy removal	4518	1818	793	468
Quantization	404354	187524	158000	41254
Huffman coding	52518	20688	9820	5557

**Fig 13:**
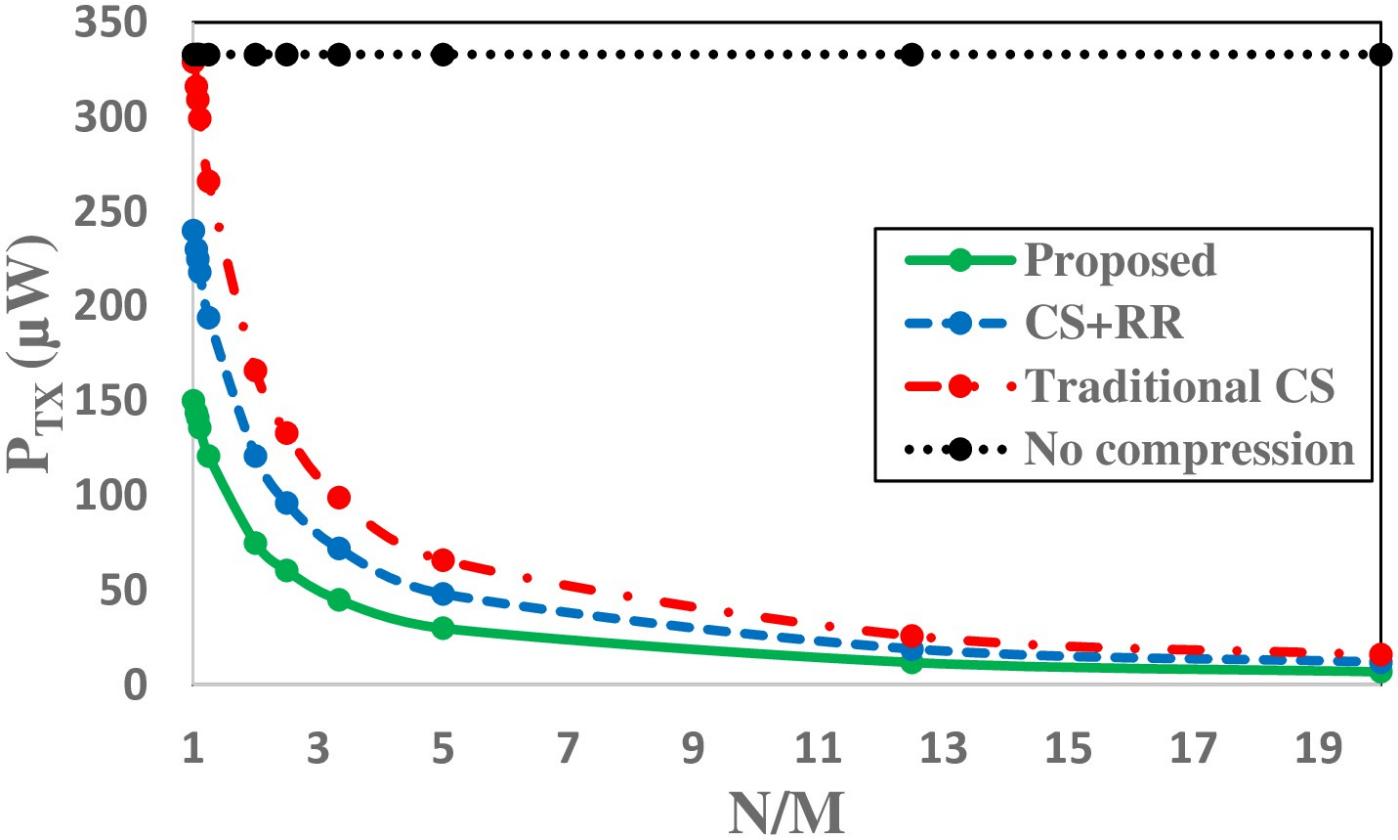
Transmission power consumption (*P*_*TX*_).

As it is obvious from Eq 8, by increasing the number of measurements, that results in an increased number of bits per frame, the energy consumed for transmitting a frame is increased, that implies the correlation between CR and transmission energy consumption. As one could see in Fig 13 because of high redundancy removed from aligned frames, the proposed technique is able to reduce power consumption in the transmission part compared to situations where ECG frames are only compressed (traditional CS [22]) and techniques in which redundancy is removed (RR) from measurements without any peak alignment (CS+ (RR)) [31, 32]. It is obvious that the proposed technique will highly reduce power consumption compared to situation where the ECG frame is going to be sent as is, without compression.

Fig 13 Shows that the proposed technique is able to save 45*μW* for transmission a single compressed frame with *N*/*M* = 2 compared to the work done in [32]. Because there is going to be a continuous transmission, for example, transmitting ECG frames of a patient for about 30 minutes, a total of 72*mW* would be saved by the proposed technique.

**Fig 14:**
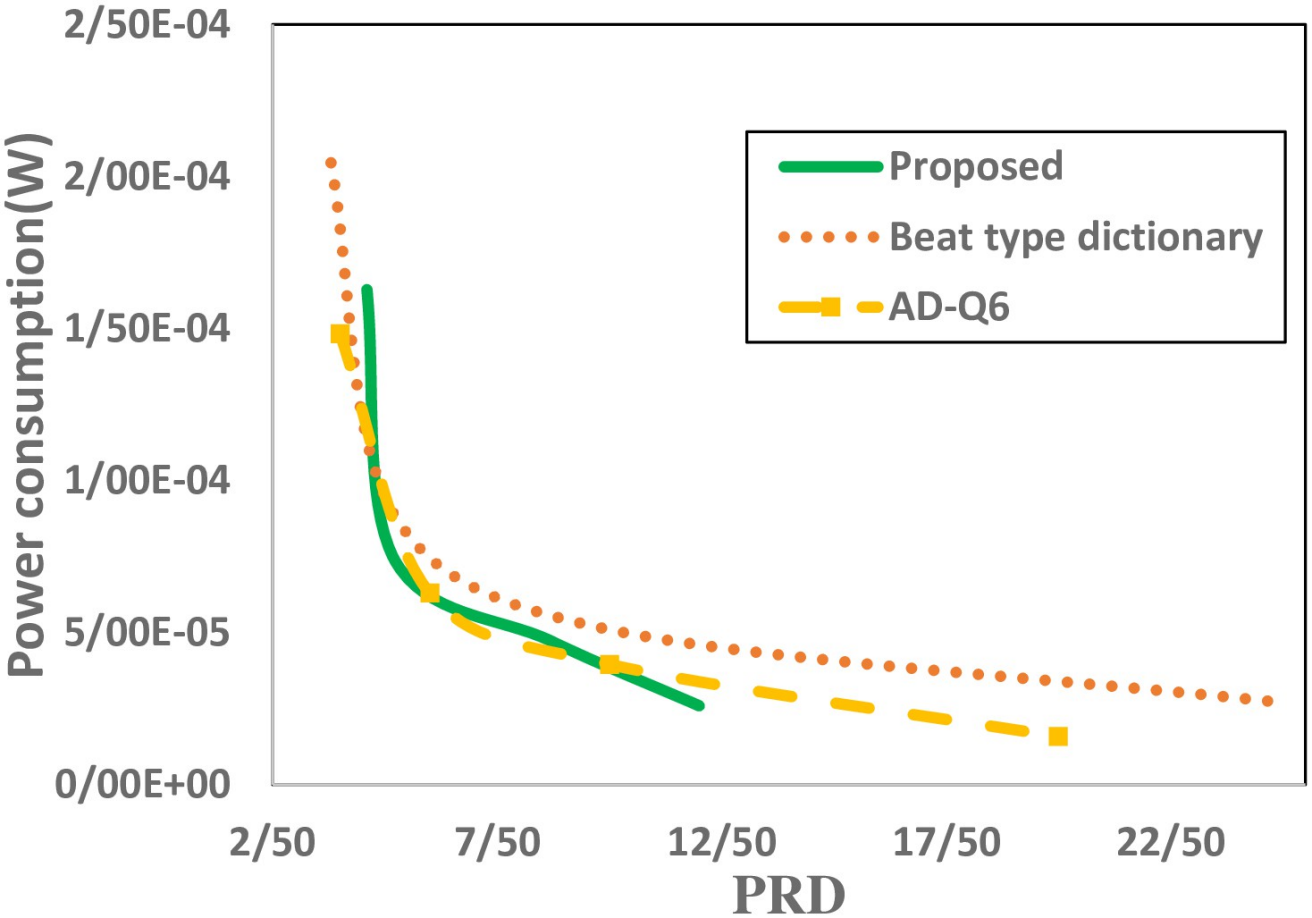
Trade-off comparison of Beat type dictionary [32], the AD-Q6 [31] and proposed work for different PRD values.

Finally in Fig 14, the energy-performance trade-off of the proposed technique is compared with the methods which improved the trade-off without aligning the ECG frames. As referred to earlier, Rakhshit et al. proposed a bit type dictionary in order to improve the performance, resulted in consuming 1.46*μW* more energy in the processing part compared to the traditional CS. Because the mentioned technique doesn’t align ECG frames before compression, like other state-of-the-art techniques, they use an 8-bit quantizer. The proposed technique, despite using a 5-bit quantizer, is able to keep the performance in an acceptable range by increasing the total energy consumption by only 3.28*μW*. As shown in Fig 14, for PRD values >5.2%, the proposed technique is the best performing algorithm. However, [32]performs best in terms of power consumption for PRDs less than 5.2%. It is clear that the efficiency of the proposed method is better than the CS techniques tested and this superiority appears to increase as the level of PRD increases.

### Effect of missed R-peak detection

Before random sensing of ECG frames, R-peaks are detected according to a technique mentioned in R-peak detection section. The peak detection technique has to be simple enough to be used in the sensor side, so the proposed peak detection technique has some errors while detecting the R-peaks. Since frames are aligned according to the detected peaks, if any peak is undetected, then the quality of the recovered ECG frame will be reduced because of the improper dictionary used. However, according to the Table 2, the percentage of R-peak detection error is very low(< 0.05%). Hence, while estimating the average quality for a long duration signal the overall quality of the recovered signal will not be affected severally.

## Conclusion

In this paper, the pseudo periodic nature of ECG signals is used to remove the hidden redundancy between measurement vectors after CS. More specifically, by canceling the redundancy from each measurement vector, a smaller quantizer could be used. An important goal of the proposed technique was reducing sensors’ energy consumption as a development challenge. The ECG records of the MIT-BIH arrhythmia database was used for simulation. The proposed work was compared with three different CS-based techniques and a wavelet-based method. The results indicate that this technique surpasses traditional CS, analog-CS and beat type dictionary in terms of PRD for *CR* > 6 and manages to reduce the gap from SPIHT and outperforms it for *CR* > 10. The proposed technique reduces power consumption in the transmission area compared to state-of-the-art techniques and only consumes 4.96*μW* more energy in the processing part compared to traditional CS. The acquired results suggest that the proposed technique is very suitable for improving the Energy-Performance trade-off of sensors for the transmission of physiological signals. Despite the performance improvements made by the proposed techniques, it has a main drawback. In wearable devices where ECG signal is highly contaminated by noise redundancy removal technique doesn’t have an effect on transmission energy consumption.

## Supporting information

S1 File (RAR)
